# Conditional promoters for analysis of essential genes in *Zymoseptoria tritici*^☆^

**DOI:** 10.1016/j.fgb.2015.03.024

**Published:** 2015-06

**Authors:** S. Kilaru, W. Ma, M. Schuster, M. Courbot, G. Steinberg

**Affiliations:** a Biosciences, University of Exeter, Exeter EX4 4QD, UK; b Syngenta Crop Protection AG, Schaffhauserstrasse, 4332 Stein, Switzerland

**Keywords:** P, promoter, *nar1*, nitrate reductase, *ex1A*, 1,4-*β*-endoxylanase, *laraB*, l-arabinofuranosidase B, *gal7*, galactose-1-phosphate uridylyltransferase 7, *icl1*, isocitrate lyase, *tub2*, α-tubulin, *sdi1*, succinate dehydrogenase 1, aa, amino acid, eGFP, enhanced green fluorescent protein, DNA, deoxyribonucleic acid, PCR, polymerase chain reaction, MT, microtubule, ROI, region of interest, Inducible–repressible promoter, Fungal wheat pathogen, Septoria tritici blotch, *Mycosphaerella graminicola*

## Abstract

Development of new fungicides, needed for sustainable control of fungal plant pathogens, requires identification of novel anti-fungal targets. Essential fungal-specific proteins are good candidates, but due to their importance, gene deletion mutants are not viable. Consequently, their cellular role often remains elusive. This hindrance can be overcome by the use of conditional mutants, where expression is controlled by an inducible/repressible promoter. Here, we introduce 5 inducible/repressible promoter systems to study essential genes in the wheat pathogen *Zymoseptoria tritici*. We fused the gene for enhanced green-fluorescent protein (*egfp*) to the promoter region of *Z. tritici* nitrate reductase (P*nar1*; induced by nitrogen and repressed by ammonium), 1,4-*β*-endoxylanase A (P*ex1A*; induced by xylose and repressed by maltodextrin), l-arabinofuranosidase B (P*laraB*; induced by arabinose and repressed by glucose), galactose-1-phosphate uridylyltransferase 7 (P*gal7*; induced by galactose and repressed by glucose) and isocitrate lyase (P*icl1*; induced by sodium acetate and repressed by glucose). This was followed by quantitative analysis of cytoplasmic reporter fluorescence under induced and repressed conditions. We show that P*nar1*, P*laraB* and P*ex1A* drive very little or no *egfp* expression when repressed, but induce moderate protein production when induced. In contrast, P*gal7* and P*icl1* show considerable *egfp* expression when repressed, and were strongly induced in the presence of their inducers. Normalising the expression levels of all promoters to that of the α-tubulin promoter P*tub2* revealed that P*laraB* was the weakest promoter (∼20% of P*tub2*), whereas P*icl1* strongly expressed the reporter (∼250% of P*tub2*). The use of these tools promises a better understanding of essential genes, which will help developing novel control strategies that protect wheat from *Z. tritici*.

## Highlights

• We establish five inducible/repressible promoters for use in *Z. tritici.*
• All promoters express cytoplasmic GFP when induced by media supplements.
• The tested conditional promoter range from weak to strong expression.
• 3 of 5 promoters are tight under repressed conditions, 2 promoters show background expression.
• A conditional α-tubulin mutant dies under repressed conditions, showing proof of principle.

## 1 Introduction

Effective management of fungal pathogens requires in-depth knowledge of their cell biology and host invasion strategies. Sophisticated sequencing methods have provided a large wealth of genes and predicted proteins, which need to be functionally analyzed to gain insight into their cellular roles. The generation of gene mutants, such as knock-out, knock-down and over-expression mutants, has greatly helped understanding protein function in eukaryotic cells (Meyer et al., 2011). Examples are the systematic deletion of genes in the budding yeast *Saccharomyces cerevisiae* (Giaever et al., 2002) and the genome-wide knock-down of proteins by RNAi silencing methods in the worm *Caenorhabditis elegans* (Kamath et al., 2003). However, a major limitation of these approaches is that cellular function of essential genes and their protein products cannot be analyzed (Meyer et al., 2011). This caveat can be overcome by the generation of conditional promoter mutants, where the expression of a gene of interest depends on the experimental condition. Such substrate-induced promoter mutants have been used in a wide range of fungi. Examples include galactose-inducible promoters of galactose-1-phosphate uridylyltransferase in *Cryptococcus neoformans* (Ruff et al., 2009), the sodium acetate inducible isocitrate lyase promoter in *Magnaporthe grisea* (Wang et al., 2003), or the arabinose-inducible, glucose repressible *crg* promoter in *Ustilago maydis* (Bottin et al., 1996). An essential requirement for conditional promoters is that they are tightly repressed in the absence of an inducer (Vu et al., 2013). On the other hand, controlled over-expression of entire genes or DNA encoding domains of interest also provides a useful way of gaining insight into their cellular role (Schuster et al., 2011).

Here, we establish 5 homologous inducible/repressible promoters in the wheat pathogen *Zymoseptoria tritici*. We identify the promoter region of nitrate reductase homologue (P*nar1*) in *Z. tritici*, which induced by nitrate and repressed by ammonium in *U. maydis* (Banks et al., 1993). Secondly, we use the promoter of l-arabinofuranosidase B homologue (P*laraB*) in *Z. tritici* that is induced by arabinose and repressed by glucose in *Aspergillus niger* (v d Veen et al., 1993). We establish use of the promoter of a 1,4-*β*-endoxylanase A homologue (P*ex1A*) in *Z. tritici*, which is induced by xylose and repressed by maltodextrin in *Aspergillus awamori* (Gouka et al., 1996). Moreover, we express eGFP under the promoter of a galactose-1-phosphate uridylyltransferase homologue (P*gal7*) in *Z. tritici*, which is induced by galactose and repressed by glucose in *C. neoformans* (Ruff et al., 2009). Finally, we use the promoter of a homologue of isocitrate lyase (P*icl1*), induced by sodium acetate and repressed by glucose in *M. grisea* (Wang et al., 2003). Using enhanced green-fluorescent protein (eGFP) as a reporter, we show that all 5 promoter systems derive eGFP expression when induced in liquid culture. Comparison with auto-fluorescence in control strains reveals that P*nar1*, P*laraB* and P*ex1A* are “shut down” under OFF conditions, whereas significant background fluorescence of eGFP was found when the reporter was placed behind P*gal7* and P*icl1*. Comparing the relative expression strength of all promoters with that of the α-tubulin promoter shows that P*laraB*, P*nar1* and P*ex1A* drive relatively weak expression under induced conditions (ON conditions), whereas P*gal7* and, in particular, P*icl1* are stronger promoters. When α-tubulin was expressed under the P*ex1A* promoter, cells grew normally, but growth was significantly impaired when P*ex1A* was repressed. This demonstrates the use of conditional promoters in functional analysis of essential genes in *Z. tritici*.

## 2 Materials and methods

### 2.1 Bacterial and fungal strains and growth conditions

*Escherichia coli* strain DH5α was used for the maintenance of plasmids. *Agrobacterium tumefaciens* strain EHA105 (Hood et al., 1993) was used for maintenance of plasmids and subsequently for *A. tumefaciens*-mediated transformation of *Z. tritici. E. coli* and *A. tumefaciens* were grown in DYT broth or DYT agar at 37 °C and 28 °C respectively. The fully sequenced *Z. tritici* wild-type isolate IPO323 (Kema and van Silfhout, 1997) was used as recipient strain for the genetic transformation experiments. The isolate was inoculated from stocks stored in glycerol at −80 °C onto solid YPD agar (yeast extract, 10 g/l; peptone, 20 g/l; glucose, 20 g/l; agar, 20 g/l) and grown at 18 °C for 4–5 days.

### 2.2 Identification of enzymes in *Z. tritici* and bioinformatics

To identify conditional promoters, we obtained the sequences of *U. maydis* Nar1 sequence (XP_759994.1), *Aspergillus fumigatus* LaraB sequence (KEY83958.1), *A. awamori* Ex1A sequence (CAA55005.1), *C. neoformans* Gal7 sequence (XP_568349.1) and *M. oryzae* Icl1 sequence (XP_003712381.1) from the NCBI server (http://www.ncbi.nlm.nih.gov/pubmed). We used these to screen the published sequence of *Z. tritici* (http://genome.jgi.doe.gov/Mycgr3/Mycgr3.home.html) using the provided BLASTP function and compared the best hits in EMBOSS Needle (http://www.ebi.ac.uk/Tools/psa/emboss_needle/). The domain structure was analyzed in PFAM (http://pfam.xfam.org/search/sequence). The promoter sequences upstream of the open reading frame were obtained from the JIG server (http://genome.jgi.doe.gov/Mycgr3/Mycgr3.home.html).

### 2.3 Molecular cloning

All vectors in this study were generated by in vivo recombination in the yeast *S. cerevisiae* DS94 (MATα, *ura3-52*, *trp1-1*, *leu2-3*, *his3-111*, and *lys2-801* (Tang et al., 1996) following published procedures (Raymond et al., 1999; Kilaru and Steinberg, 2015). For all the recombination events, the fragments were amplified with 30 bp homologous sequences to the upstream and downstream of the fragments to be cloned (see Table 1 for primer details). PCR reactions and other molecular techniques followed standard protocols (Sambrook and Russell, 2001). All restriction enzymes and reagents were obtained from New England Biolabs Inc (NEB, Herts, UK).

### 2.4 Construction of vectors pCP*nar1*eGFP, pCP*ex1*eGFP, pCP*laraB*-eGFP*,* pCP*gal7*-eGFP*, p*CP*icl1*-eGFP and pH*ex1A*-tub2

Vector pCP*Pnar1*eGFP contains *egfp* under the control of *Z. tritici nar1* promoter for integration into the *sdi1* locus by using carboxin as a selectable marker. A 13,083 bp fragment of pCeGFPTub2 (digested with *Pml*I; Schuster et al., 2015) and 1000 bp *Z. tritici nar1* promoter (amplified with SK-Sep-112 and SK-Sep-113; Table 1) were recombined in *S. cerevisiae* to obtain the vector pCP*nar1*eGFP. Vector pCP*ex1*eGFP contains *egfp* under the control of *Z. tritici ex1A* promoter for integration into the *sdi1* locus by using carboxin as a selectable marker. A 13,083 bp fragment of pCeGFPTub2 (digested with *Pml*I) and 1000 bp *Z. tritici ex1A* promoter (amplified with SK-Sep-114 and SK-Sep-115; Table 1) were recombined in *S. cerevisiae* to obtain the vector pC*Pex1*eGFP. Vector pCP*laraB*eGFP contains *egfp* under the control of *Z. tritici laraB* promoter for integration into the *sdi1* locus by using carboxin as a selectable marker. A 13,083 bp fragment of pCeGFPTub2 (digested with *Pml*I) and 1,000 bp *laraB* promoter (amplified with SK-Sep-118 and SK-Sep-119; Table 1) were recombined in *S. cerevisiae* to obtain the vector pCP*laraB*eGFP. Vector pCP*gal7*-eGFP contains *egfp* under the control of *Z. tritici gal7* promoter for integration into the *sdi1* locus by using carboxin as a selectable marker. A 13,083 bp fragment of pCeGFPTub2 (digested with *Pml*I) and 1000 bp *Z. tritici gal7* promoter (amplified with SK-Sep-126 and SK-Sep-127; Table 1) were recombined in *S. cerevisiae* to obtain the vector pCP*gal7*-eGFP. Vector pCP*icl1*-eGFP contains *egfp* under the control of *Z. tritici icl1* promoter for integration into the *sdi1* locus by using carboxin as a selectable marker. A 13,083 bp fragment of pCeGFPTub2 (digested with *Pml*I) and 1000 bp *Z. tritici icl1* promoter (amplified with SK-Sep-120 and SK-Sep-121; Table 1) were recombined in *S. cerevisiae* to obtain the vector pCP*icl1*-eGFP. Vector pHP*ex1A*Tub2 contains the *Z. tritici ex1A* promoter (*Pex1A*) fused to *tub2* gene for replacement of endogenous *tub2* promoter in *Z. tritici* with *ex1A* promoter using hygromycin as a selectable marker. A 9533 bp fragment of pCeGFPTub2 (digested with *Bam*HI and *Hind*III), 1149 bp *tub2* promoter (amplified with SK-Sep-217 and SK-Sep-218; Table 1), 1806 bp hygromycin resistance cassette (amplified with SK-Sep-136 and SK-Sep-137; Table 1), 1000 bp *ex1A* promoter (amplified with SK-Sep-225 and SK-Sep-226; Table 1), 5′ end of 1002 bp *tub2* gene (amplified with SK-Sep-221 and SK-Sep-222; Table 1) were recombined in *S. cerevisiae* to obtain the vector pHP*ex1A*tub2.

### 2.5 *Z. tritici* transformation

The vectors pCP*nar1*eGFP, pCP*ex1*eGFP and pCP*laraB*eGFP, pCP*gal7*eGFP and pCP*icl1*eGFP were transformed into *A. tumefaciens* strain EHA105 by heat shock method (Holsters et al., 1978), following previously published protocols (Zwiers and De Waard, 2001). Further details on this method are provided in Kilaru et al. (2015a).

### 2.6 *Z. tritici* transformation of vector pHP*ex1A*Tub2

The plasmid pHP*ex1A*Tub2 was transformed to *Z. tritici* isolate IPO323 as described in Kilaru et al. (2015a) with few modifications resulting in strain IPO323_P*ex1A*Tub2. In order to ensure that *ex1A* promoter is ON, 5% xylose (Sigma–Aldrich, Gillingham, UK) was added to *Agrobacterium* induction medium agar plates. For selection of transformants, 200 μg/ml hygromycin (Roche, Burgess Hill, UK) was added to the Czapekdox agar containing 5% xylose, along with100 μg/ml cefotaxime and 100 μg/ml timentin. The individual transformants were transferred to complete medium CM (casaminoacids, 2.5 g/l; yeast extract. 1.0 g/l; NH4NO3, 1.5 g/l; DNA from herring sperm, 0.5 g/l; salt solution, 62.5 ml/l; vitamin solution 10.0 ml/l; pH-7.0; Holliday, 1974) with 5% xylose, 200 μg/ml hygromycin, 100 μg/ml cefotaxime and 100 μg/ml timentin and grown at 18 °C for 3–4 days.

### 2.7 Molecular analysis of transformants

To confirm the integration of vectors pCP*nar1*eGFP, pCP*ex1*eGFP, pCP*laraB*eGFP, pCP*gal7*eGFP and pCP*icl1*eGFP into the *sdi1* locus of *Z. tritici*, Southern blot hybridizations were performed by using the standard procedures (Sambrook and Russell, 2001). *Z. tritici* wild-type IPO323 and transformants obtained with vectors pCP*nar1*eGFP, pCP*ex1*eGFP, pCP*laraB*eGFP, pCP*gal7*eGFP and pCP*icl1*eGFP were grown in YG broth (yeast extract, 10 g/l; glucose 30 g/l) for 3 days at 18 °C with 200 rpm. 3 ml of yeast-like cells were used for fungal genomic DNA extraction as described (Kilaru et al., 2015a). Approximately 3 μg of genomic DNA of IPO323 and transformants obtained with vectors pCP*nar1*eGFP, pCP*ex1*eGFP, pCP*laraB*eGFP, pCP*gal7*eGFP and pCP*icl1*eGFP were digested with *Bgl*II and separated on a 1.0% agarose gel and capillary transferred to a Hybond-N membrane (GE healthcare, Little Chalfont, United Kingdom). 1014 bp *sdi1* probe (3′ end of the *sdi1^R^* gene and *sdi1* terminator) was generated by using DIG labeling PCR mix (Life Science Technologies, Paisley, UK) with primers SK-Sep-10 and SK-Sep-13; Table 1. Hybridizations were performed at 62 °C for overnight autoradiographs were developed after an appropriate time period.

Integration of vector pHP*ex1A*Tub2 into the *tub2* locus of *Z. tritici* was confirmed by Southern blot*. Z. tritici* transformants obtained with plasmid pHP*ex1A*Tub2 were grown in complete medium CM with 5% xylose for 3 days at 18 °C with 200 rpm. Approximately 3 μg of genomic DNA of IPO323 and transformants obtained with vector pHP*ex1A*Tub2 were digested with *Sac*II, and separated on a 1.0% agarose gel and capillary transferred to a Hybond-N membrane (GE healthcare, Little Chalfont, United Kingdom). 1149 bp *tub2* probe (α-tubulin promoter) was generated by using DIG labeling PCR mix (Life Science Technologies, Paisley, UK) with primers SK-Sep-217 and SK-Sep-218; Table 1. Hybridizations were performed at 62 °C for overnight autoradiographs were developed after an appropriate time period.

### 2.8 Investigation of eGFP expression under ON- and OF conditions

IPO323_CP*nar1*eGFP cells were grown in Nitrate minimal medium NM (KNO3, 3.0 g/l; salt solution, 62.5 ml/l; pH-7.0; Holliday, 1974) with 1% glucose (ON) and Complete medium (CM) with 1% glucose (OFF); the IPO323_CP*ex1A*eGFP cells were grown in *Aspergillus* minimal medium (NaNO_3_, 6 g/l; KCl, 0.52 g/l; KH_2_PO_4_, 1.52 g/l; MgSO_4_·7H_2_O, 0.52 g/l and trace elements solution 1 ml; pH-6.5, Kafer, 1977) with 5% xylose (ON) and *Aspergillus* minimal medium with 5% maltodextrin (OFF); the IPO323_CP*laraB*eGFP cells were grown in *Aspergillus* minimal medium with 2% arabinose (ON) and *Aspergillus* minimal medium with 1% glucose (OFF); the IPO323_CP*gal7*eGFP cells were grown in *Aspergillus* minimal medium with 0.5% galactose (ON) and *Aspergillus* minimal medium with 2% glucose (OFF); the IPO323_CP*icl1*eGFP cells were grown in *Aspergillus* minimal medium with 20 mM sodium acetate (ON) and *Aspergillus* minimal medium with 2% glucose (OFF) at 18 °C with 200 rpm for 24 h. In order to compare the expression levels of eGFP in strains IPO323_CP*nar1*eGFP, IPO323_CP*ex1A*eGFP, IPO323_CP*laraB*eGFP, IPO323_CP*gal7*eGFP and IPO323_CP*icl1*eGFP with eGFP expression under the *tub2* promoter in strain IPO323_CeGFP (Kilaru et al., 2015a). Strain IPO323_CeGFP was used in all corresponding control experiments.

Fluorescence microscopy was performed as previously described (Schuster et al., 2011). In brief, cells were placed onto a 2% agar cushion for direct observation using a motorized inverted microscope (IX81; Olympus, Hamburg, Germany), equipped with a PlanApo 100×/1.45 Oil TIRF (Olympus, Hamburg, Germany). The expression of eGFP under the different inducible/repressible promoters and *tub2* promoter was analyzed by capturing single images with a 150 ms exposure time using a CoolSNAP HQ2 camera (Photometrics/Roper Scientific, Tucson, USA). The fluorescent tags of all strains, with the exception of IPO323_P*icl1*eGFP, were excited using a VS-LMS4 Laser Merge System with a 488 nm solid-state laser (75 mW; Visitron Systems, Puchheim, Germany) at 20%. IPO323_P*icl1*eGFP was excited with 10% of the 488 nm solid-state laser to avoid camera saturation.

Average intensity was analyzed in those images by creating a region of interest (ROI) within a cell covering only a part of the cytoplasm but excluding the nucleus or vacuoles. A copy of the same ROI was placed next to the cell to get the average intensity of the neighbouring background. Both values were transferred to Excel (Microsoft, Redmond, WA, USA) and the value of the background was subtracted from the value of the cell. Those corrected values were transferred to Prism 4.03 (GraphPad Software, La Jolla, CA, USA) to perform all statistical analysis. All parts of the system were under the control of the software package MetaMorph (Molecular Devices, Wokingham, UK).

### 2.9 Plate growth assay

The complete medium CM agar either with 5% xylose (Sigma–Aldrich, Gillingham, UK) or 5% maltodextrin (Sigma–Aldrich, Gillingham, UK) was used to examine the growth habit of IPO323 and IPO323_HP*ex1A*Tub2. For better visualisation of the colonies, 1% activated charcoal was added to the media. The IPO323 and IPO323_HP*ex1A*Tub2 cells were grown in CM with 5% xylose at 18 °C with 200 rpm for 2 days. The cell density was adjusted to an optical density of 0.6 at 660 nm. 1,000 μl of cell culture was transferred to 1.5 ml Eppendorf tube and centrifuged at 3000 rpm for 3 min. The supernatant was discarded and the cell pellet was resuspended either with CM containing 5% xylose (ON condition) or 5% maltodextrin (OFF condition). The cell cultures were serially diluted (10 times each) with the corresponding media. The serial diluted cultures were then applied as 5 μl droplets on either CM agar with 5% xylose and 1% charcoal or CM agar with 5% maltodextrin and 1% charcoal and grown at 18 °C for 6 days. Photographs of the relative colony densities were taken using a canon digital IXUS 80 IS camera (Canon, Surrey, UK).

## 3 Results and discussion

### 3.1 Identification of homologues of nitrate reductase, of l-arabinofuranosidase B, 1,4-*β*-endoxylanase A, galactose-1-phosphate uridylyltransferase and isocitrate lyase in *Z. tritici*

The *Z. tritici* homologues of the *U. maydis* nitrate reductase (Nar1), *A. fumigatus* l-arabinofuranosidase B (LaraB) and *A. awamori* 1,4-*β-*endoxylanase A (Ex1A), *C. neoformans* galactose-1-phosphate uridylyltransferase (Gal7) and *M. oryzae* isocitrate lyase (Icl1) were identified by BLASTP search (http://blast.ncbi.nlm.nih.gov/Blast.cgi) with the *U. maydis* Nar1 sequence (XP_759994.1), *A. fumigatus* LaraB sequence (KEY83958.1), *A. awamori* Ex1A sequence (CAA55005.1), *C. neoformans* Gal7 sequence (XP_568349.1) and *M. oryzae* Icl1 sequence (XP_003712381.1) against the *Z. tritici* published genome sequence of IPO323 (Kema and van Silfhout, 1997) at the Joint Genome Institute (http://genome.jgi.doe.gov/Mycgr3/Mycgr3.home.html). Most similar to *U. maydis* Nar1 *in Z. tritici* was protein 111003 (NCBI accession number: XP_003849160.1), to *A. fumigatus* LaraB in *Z. tritici* was protein 70396 (NCBI accession number: XP_003854224.1), to *A. awamori* Ex1A in *Z. tritici* was protein 60105 (NCBI accession number: XP XP_003851797.1), to *C. neoformans* Gal7 in *Z. tritici* was protein 72281 (NCBI accession number: XP_003852576.1) and to *M. oryzae* Icl1 in *Z. tritici* was protein 102083 (XP_003855965.1), respectively. The predicted proteins ZtNar1, ZtLaraB, ZtEx1A, ZtGal7 and ZtIcl1 shared 39.6%, 45.3%, 40.4%, 42.0% and 71.7% aa identity with their homologues and have a similar domain structure. ZtNar1 shares with Nar1 from *U. maydis* an oxidoreductase molybdopterin binding domain (*P* = 8.6e−49), a molybdopterin cofactor oxidoreductase dimerisation domain (*P* = 8.6e−4), a cytochrome b5-like heme/steroid binding domain (*P* = 3.8e-23), an oxidoreductase FAD-binding domain (*P* = 3.5e−31) and an oxidoreductase NAD-binding domain (*P* = 6.4e−05). ZtLaraB shares with LaraB from *A. fumigatus* an alpha-l-arabinofuranosidase B domain (*P* = 2.1e−132), and ZtEx1A shares with Ex1A from *A*. *awamori* a glycosyl hydrolases family 11 domain (*P* = 4.6e−83). ZtGal7 shares with Gal7 from *C. neoformans* two Galactose-1-phosphate uridyl transferase domains (*P* = 2.6e−68, 1.6e−55) and ZtIcl1 shares an isocitrate lyase family domain with Icl1 from *M. oryzae* (*P* = 1.7e−278).

### 3.2 Vectors for targeted ectopic integration of eGFP, expressed under the promoters P*nar1*, P*ex1A*, P*laraB*, P*gal7* and P*icl1*

**Fig. 1:**
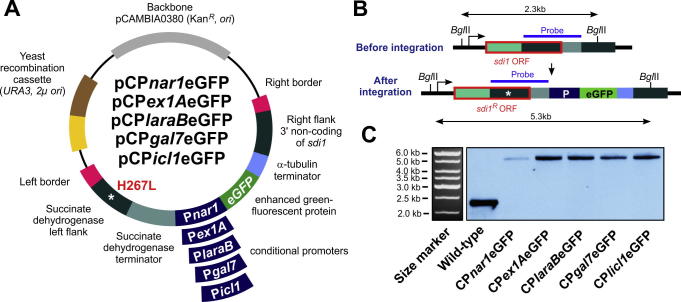
Vectors for succinate dehydrogenase (*sdi1*) locus integration that allow expression of enhanced GFP under inducible/repressible promoters. (A) Organization of three vectors that allow enhanced GFP expression after induction of inducible/repressible promoters P*nar1* (promoter region of a *Z. tritici* homologue of nitrate reductase; NCBI accession number, XP_003849160.1), P*ex1A* (promoter region of a *Z. tritici* homologue of 1,4-*b*-endoxylanase A), P*laraB* (promoter region of a *Z. tritici* homologue of l-arabinofuranosidase B), P*gal7* (promoter region of a *Z. tritici* homologue of galactose-1-phosphate uridylyltransferase) and P*icl1* (promoter region of a *Z. tritici* homologue of isocitrate lyase). After integration into the *sdi1* locus, the vector confers carboxin resistance due to a point mutation in the succinate dehydrogenase gene *sdi1*, which changes a histidine to a leucine (H267L). For more details of this integration into the “succinate dehydrogenase locus” see Kilaru et al., 2015a. Left and right border enable *A. tumefaciens*-based transformation of *Z. tritici*. (B) Diagram showing the organization of the *sdi1* locus before and after integration of the GFP-encoding vectors. Note that integration of the point mutated *sdi1* left flank (see Fig. 1A; point mutation indicated by asterisk) replaces a part of the *sdi1* open reading frame (*sdi1* ORF) and confers carboxin resistance (*sdi1^R^* ORF). Successful integration of the vector increases the size of a DNA fragment after digestion with the restriction enzyme *Bgl*II and subsequent detection with a labelled DNA probe (blue bar). (C) Southern blot, showing integration of the described vectors into the *sdi1* locus. After digestion of the genomic DNA with *Bgl*II and subsequent hybridisation with a labelled DNA probe, a shift in the DNA fragment from 2.3 kb to 5.3 kb is detected. The size markers in the corresponding agarose gel are shown to the left.

We next defined the promoter region upstream of each open reading frame (P*nar1*, P*ex1A*, P*laraB*, P*gal7* and P*icl1*). We choose 1000 bp, which includes the majority of putative promoter sequences in the yeast *S. cerevisiae* (Kristiansson et al., 2009). To test if these putative promoter regions are inducible/repressible, we generated 5 vectors (pCP*nar1*eGFP, pCP*laraB*eGFP, pCP*ex1Ae*GFP, pCP*gal7e*GFP and pCP*icl1e*GFP) that drive expression of eGFPs in *Z. tritici*. All vectors were built on the *Agrobacterium* binary vector pCAMBIA0380 (CAMBIA, Canberra, Australia) to enable *A. tumefaciens*-based transformation into *Z. tritici*, which is based on the 25 bp imperfect directional repeat sequences of the T-DNA borders (right and left border, RB and LB; Fig. 1A). In addition, all three vectors comprise a “yeast recombination cassette”, allowing yeast recombination-based cloning (for more details see Kilaru and Steinberg, 2015). Finally, we designed all vectors for targeted integration into the *sdi1* locus of *Z. tritici* genome, by using a mutated downstream stretch of the *sdi1* sequence, carrying a carboxin resistance conferring point mutation (H267L; Fig. 1A, left flank), and a sequence stretch downstream of *sdi1* (Fig. 1A, right flank of *sdi1*). Incorporation by homologous recombination mutates the *sdi1* gene and integrates the eGFP constructs into the *sdi1* locus (for details see Kilaru et al., 2015a). Thus, all 5 constructs allow eGFP expression in an identical genomic environment and single integration of each construct, which is essential for quantitative analysis of fluorescent intensities.

We next transformed all 5 vectors into *Z. tritici* wild-type IPO323 (Kema and van Silfhout, 1997), using *A. tumefaciens*-mediated transformation (Zwiers and De Waard, 2001). In order to confirm the single copy integration into the *sdi1* locus, we purified genomic DNA from the transformants and wild-type isolate IPO323, digested with *Bgl*II and hybridized this to an *sdi1* probe (Fig. 1B). Indeed, integration of the respective vectors resulted in shifting of a single band at the expected sizes (from 2.3 to 5.3 kb, Fig. 1B and C; marker bands in corresponding agarose gel are shown in Fig. 1C). This confirmed that the P*nar1*, P*laraB,* P*ex1A,* P*gal7 and* P*icl1* promoters and the downstream e*gfp* were integrated correctly into the *sdi1* locus as single copies. We took single transformants of each strain and named them IPO323_CP*nar1*eGFP, IPO323_CP*laraB*eGFP, IPO323_CP*ex1A*eGFP, IPO323_CP*gal7*eGFP and IPO323_CP*icl1*eGFP. All strains contained a single promoter-*gfp* reporter construct in their *sdi1* locus, which allowed quantitative comparison of the cytoplasmic eGFP signals as a reporter for *egfp* gene expression.

### 3.3 P*nar1*, P*laraB,* P*ex1A,* P*gal7* and P*icl1* controlled eGFP expression under induced and repressed conditions

**Fig. 2:**
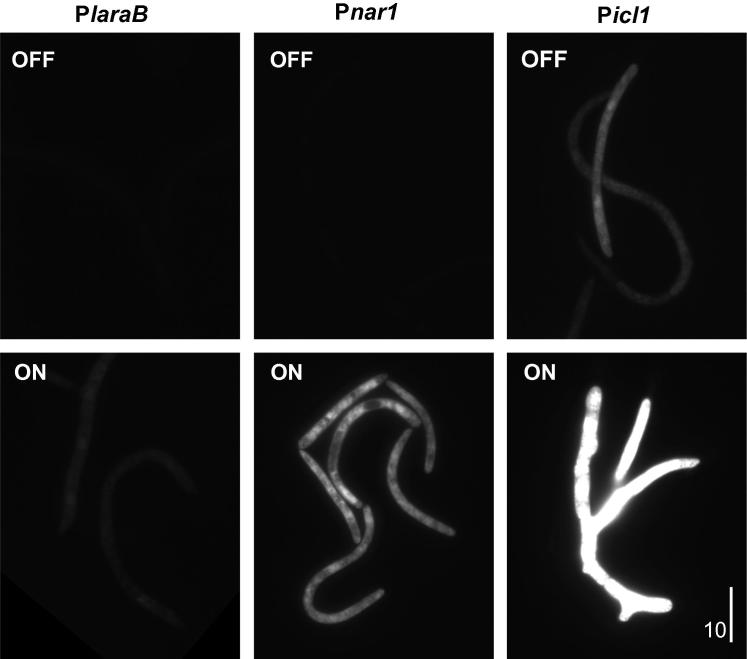
Expression of cytoplasmic eGFP under inducible/repressible conditions. Images showing eGFP fluorescence under repressive (OFF) and induced conditions (ON). Note that virtually no signal is detected when P*laraB* and P*nar* repressed, whereas weak fluorescence appears when P*icl1* is repressed. All strains show fluorescence after induction, but the intensity of the signal varies remarkably. Note that all images are processed identically. Bar represents 10 μm.

**Fig. 3:**
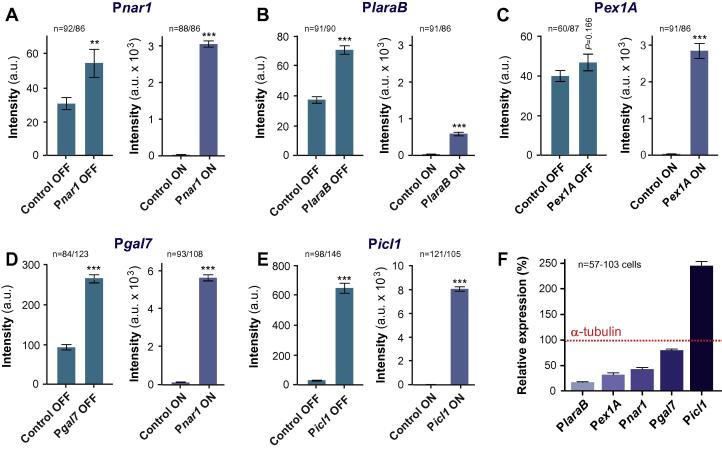
Quantitative analysis of eGFP expression under inducible/repressible conditions. (A) Bar charts showing the extent of cytoplasmic fluorescence of eGFP in control cells and IPO323_CP*nar1*-eGFP in their *sdi1* locus, both grown under repressed (OFF) and induced (ON) conditions. Mean ± standard error of the mean is shown, sample size *n* represents cells and is indicated. Double asterisk indicates significant difference at *P* = 0.004; triple asterisk indicates significant difference at *P* < 0.0001, Student *t*-test. Note that the data set represent measurements from two experiments and a single transformant. (B) Bar charts showing the extent of cytoplasmic fluorescence of eGFP in control cells and IPO323_CP*laraB*-eGFP cells, both grown under repressed (OFF) and induced (ON) conditions. Mean ± standard error of the mean is shown, sample size *n* represents cells and is indicated. Triple asterisk indicates significant difference at *P* < 0.0001, Student *t*-test. Note that the data set represent measurements from two experiments and a single transformant. (C) Bar charts showing the extent of cytoplasmic fluorescence of eGFP in control cells and IPO323_CP*ex1A*eGFP cells, both grown under repressed (OFF) and induced (ON) conditions. Mean ± standard error of the mean is shown, sample size *n* represents cells and is indicated. Triple asterisk indicates significant difference at *P* < 0.0001, Student *t*-test. Note that no green fluorescence was detected when expression of eGFP was controlled by the 1,4-*β*-endoxylanase A promoter (P*ex1A*; no difference to auto-fluorescence at *P* = 0.166; Student *t*-test). Note that the data set represent measurements from two experiments and a single transformant. (D) Bar charts showing the extent of cytoplasmic fluorescence of eGFP in control cells and IPO323_CP*gal7*eGFP, both grown under repressed (OFF) and induced (ON) conditions. Mean ± standard error of the mean is shown, sample size *n* represents cells and is indicated. Triple asterisks indicate significant difference at *P* < 0.0001, Student *t*-test. Note that the promoter allows weak expression of eGFP under repressed conditions. Note also that the data set represent measurements from two experiments and a single transformant. (E) Bar charts showing the extent of cytoplasmic fluorescence of eGFP in control cells and IPO323_CP*icl1*eGFP, both grown under repressed (OFF) and induced (ON) conditions. Mean ± standard error of the mean is shown, sample size *n* represents cells and is indicated. Triple asterisks indicate significant difference at *P* < 0.0001, Student *t*-test. Note that the promoter allows considerable expression of eGFP under repressed conditions. Note also that the data set represent measurements from two experiments and a single transformant. (F) Bar chart showing the relative cytoplasmic fluorescence of eGFP in all promoter strains under induced conditions. All intensities were normalised against eGFP fluorescence, driven by the α-tubulin promoter P*tub2*.

To test if P*nar1*, P*laraB*, P*ex1A,* P*gal7 and* P*icl1* are substrate-repressible, we grew liquid cultures of strains IPO323_CP*nar1*eGFP, IPO323_CP*laraB*eGFP, IPO323_CP*ex1A*eGFP, IPO323_CP*gal7*eGFP and IPO323_CP*icl1*eGFP for 24 h in the presence of the respective repressor (P*nar1*: 20 mM ammonium; P*laraB*: 1% glucose; P*ex1A:* 5% maltodextrin; P*gal7*: 2% glucose; P*icl1*: 2% glucose). Under these conditions, almost no cytoplasmic fluorescence was observed for P*nar1*, P*laraB* and P*ex1A* (Fig. 2, OFF; only P*laraB* and P*nar1* shown; 3A–C, OFF). Notably, P*ex1A*-*egfp* containing cells showed a signal intensity that was not significantly higher than that of untransformed IPO323 (Control; Fig. 3C; *P* = 0.166, Student *t*-test), suggesting that the promoter is “tight” under repressed conditions. In contrast, P*gal7* and P*icl1* were not repressed entirely and weak GFP fluorescence was still visible under OFF conditions (Fig. 2, OFF; only P*icl1* is shown; Fig. 3D and E, OFF). After 24 h growth of cells in the media supplemented with their respective inducers (P*nar1*: 30 mM nitrate; P*laraB*: 2% arabinose; P*ex1A:* 5% xylose; P*gal7*: 0.5% galactose; P*icl1*: 20 mM sodium acetate), all strains showed cytoplasmic fluorescence, indicating eGFP expression from the induced promoters (Figs. 2 and 3A–E, ON). However, induction levels varied between the three promoters. P*nar1* and P*ex1A* were induced ∼127 and ∼415-times when compared to repressed conditions, respectively, whereas P*icl1*, P*laraB* and P*gal7* expression was only induced ∼13-, ∼17- and ∼31-times, respectively (Fig. 3, ON). These numbers correspond well with published results in other fungi, where the *nar1* promoter in *U. maydis* is induced 90-times (Banks et al., 1993), the *laraB* promoter in *A. fumigatus* is induced 13.7-times (v d Veen et al., 1993), the *A. awamori* 1,4-*β*-endoxylanase A promoter is induced 1346-times (Gouka et al., 1996), the *gal7* promoter in *C. neoformans* is induced by 21.6-times (Ruff et al., 2009) and the *icl1* promoter in *M. oryzae* is induced by 6-times (Wang et al., 2003).

We noticed that the fluorescent signal intensity of cytoplasmic eGFP varied remarkably when the reporter was expressed under the various promoters (Fig. 2). We tested the “relative expression strength” of all 5 promoters by comparing their eGFP expression with that of the α-tubulin promoter P*tub2*, which was also integrated into the *sdi1* locus of IPO323 (strain IPO323_CP*tub2*eGFP; further details on this strain (named as IPO323_CeGFP) in Kilaru et al., 2015b). We found that P*laraB* was shows the weakest relative expression level when induced (∼20% of P*tub2*, Fig. 3F; Table 2). P*ex1A*, P*nar1*, and P*gal7* were stronger than P*laraB*, but weaker than P*tub2* (∼32%, ∼43%, ∼80%, respectively; Fig. 3F, Table 2). P*icl1* induced very strong expression of eGFP (∼250% of P*tub2*, Fig. 3F; Table 2).

### 3.4 A conditional α-tubulin mutant in *Z. tritici*

**Fig. 4:**
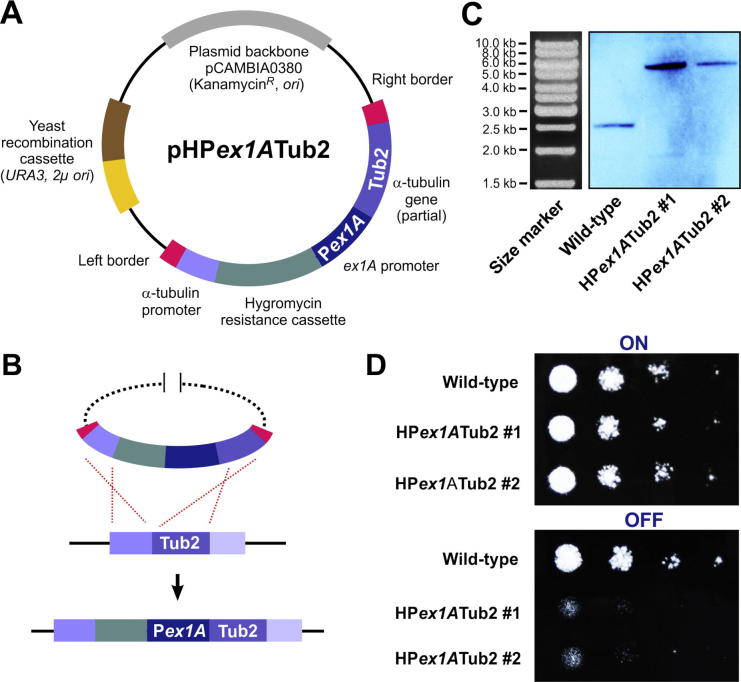
A conditional α-tubulin mutant in *Z. tritici*. (A) Vector for homologous integration of the inducible/repressible promoter *Pex1A* in front of the open reading frame of the *Z. tritici* α-tubulin gene *tub2* (see Schuster et al., 2015, for more information on *tub2*). Note that fragments are not drawn to scale. See main text for further information. (B) Image illustrates the integration event of vector pHP*ex1A*Tub2 into the native *tub2* locus of *Z. titici*. After integration, expression of the endogenous α-tubulin gene is under the control of inducible/repressible promoter. (C) Southern blot, showing integration of vector into the *tub2* locus in two transformants. After digestion of the genomic DNA with *Sac*II and subsequent hybridisation with a DIG labelled DNA probe, a shift in the DNA fragment from 2.6 kb to 5.4 kb is detected. The size markers in the corresponding agarose gel are shown to the left. (D) Growth of conditional *tub2* mutants on agar plates supplemented with 5% xylose (induces expression, ON) and 5% maltodextrin (represses expression, OFF). α-tubulin is an essential gene and, consequently, colonies cannot be formed when *tub2* expression is repressed. Dilution steps are 10 times starting with cell density of optical density of 0.6.

P*ex1A* was most “tight” under repressed condition, suggesting that it is best suited to investigate the effect of repressing expression of essential genes. We set out to provide “proof of principle”, by expressing the α-tubulin gene *tub2* und the control of P*ex1A*. The predicted gene product ZtTub2 shows 77.4%*,* aa sequence identity with Tub1 from *U. maydis* (see Schuster et al., 2015, for more details). We generated plasmid pHP*ex1A*Tub2, which carries the 5′ end of *tub2* fused to P*ex1A* (Fig. 4A) and was designed to introduce the conditional promoter into the native *tub2* locus of *Z. tritici* (Fig. 4B shows integration of P*ex1A*Tub2 into *tub2* locus). Similar to the plasmids described above, pHP*ex1A*Tub2 was built into the *Agrobacterium* binary vector pCAMBIA0380 (CAMBIA, Canberra, Australia) for *A. tumefaciens*-based transformation and is suited for yeast recombination-based cloning (for more details on this method see Kilaru and Steinberg, 2015). We transformed vector pHP*ex1A*Tub2 into *Z. tritici* wild-type strain IPO323 and confirmed integration into the *tub2* locus by digestion of genomic DNA with *Sac*II, which shifted a fragment, detected by hybridization to a *tub2* probe, from 2.6 kb to 5.4 kb (Fig. 4C; 2 transformants shown). This confirmed that the endogenous *tub2* promoter in *Z. tritici* was replaced with *ex1A* promoter, resulting in strain IPO323_HP*ex1A*Tub2. α-Tubulin is an essential protein, required to form microtubules and the mitotic spindle (Schatz et al., 1988). We therefore expected that repression of P*ex1A* would inhibit growth. We tested this by growing IPO323 (Fig. 4D, Wild-type) and two mutant strains (Fig. 4D, IPO323_HP*ex1A*Tub2#1, IPO323_HP*ex1A*Tub2#2) on agar medium containing 5% xylose. Under these conditions, *tub2* expression was induced and cells grew normally. However, when colonies were cultivated on 5% maltodextrin-containing agar medium, expression of *tub2* was repressed in both mutants and cells did not grow, whereas IPO323 was not affected (Fig. 4D). It is worth noting that while P*ex1A* repression appears to be relatively “tight” and repressing α-tubulin under this promoter impairs growth, other genes of interest that may have low native expression levels may not be sufficiently repressed under these conditions. However, these results show that conditional promoters can be useful tools to study the importance of essential genes in *Z. tritici*.

## 4 Conclusion

In this study we established 5 conditional promoters for use in *Z. tritici*. This adds a repertoire of homologous promoters to the established heterologous isocitrate lyase promoter derived from *N. crassa* (Rohel et al., 2001). Using eGFP as a reporter we show that all promoters can be repressed under specific conditions, but allow expression in media supplemented with respective inducers. Having a choice of 5, one may ask which promoter is best suited for use in *Z. tritici*? P*nar1*, P*laraB*, and P*ex1A* are relatively “tight” when their repressors are present (Table 2), suggesting that they are suitable to investigate the cellular roles of essential genes. Moreover, all three promoters drive relatively weak expression under induced conditions, which reduces the risk of over-expression artifacts when essential genes are under their control. However, depending on the native expression level of a gene of interest, P*gal7* may be a better choice, as its expression level under ON conditions is significantly higher than that of P*laraB*, P*nar1* and P*ex1A*, while the promoter is still clearly repressed in the presence of glucose. Furthermore, experimental conditions could dictate the use of a specific promoter. For example, analysis of sugar metabolic pathways precludes the usage of P*laraB*, but suggests the usage of P*nar1*. On the other hand, P*ex1A* is the tightest promoter, as it shows no eGFP fluorescence, suggesting that eGFP expression is strongly repressed under OFF-conditions. Therefore, this promoter may be useful in investing signaling cascade components, which could act in very low concentrations in the cell and, therefore, require a near-complete shutdown of their transcription. P*laraB*, on the other hand, shows relatively weak expression under induced conditions. This could be useful to avoid over-expression of genes that have naturally low cellular expression. Finally, P*icl1* showed exceptionally strong expression under induced conditions, but was not tightly “shut-down” in the presence of its repressor. Thus, P*icl1* may be a useful tool for over-expression studies, which, again, will depend on the native expression level of the genes of interest. Taken together, the set of conditional promoters are a powerful tool to perform in-depth analysis of essential genes in *Z. tritici*.

## Figures and Tables

**Table 1:** Primers used in this study.

Primer name	Direction	Sequence (5′ to 3′)^a^
SK-Sep-10	Sense	*TGGCAGGATATATTGTGGTGTAAACAAATT*GACCTTCCACATCTACCGATGG
SK-Sep-13	Antisense	CTTCCGTCGATTTCGAGACAGC
SK-Sep-14	Sense	*CATTTGCGGCTGTCTCGAAATCGACGGAAG*GCAGTCGACGCCAGATGATGG
SK-Sep-15	Antisense	*GGTGAACAGCTCCTCGCCCTTGCTCACCAT*GGCGATGGTGGTATGCGGATG
SK-Sep-112	Sense	*CATTTGCGGCTGTCTCGAAATCGACGGAAG*ATAGTTGCTCTACGACCAATGCC
SK-Sep-113	Antisense	*GGTGAACAGCTCCTCGCCCTTGCTCACCAT*TGCGGGAGAGGACATAGTAACG
SK-Sep-114	Sense	*CATTTGCGGCTGTCTCGAAATCGACGGAAG*GGGAGAGCTTGCCCGCTGGC
SK-Sep-115	Antisense	*GGTGAACAGCTCCTCGCCCTTGCTCACCAT*GACTTTGTTTGATCTCGAAGCTGA
SK-Sep-118	Sense	*CATTTGCGGCTGTCTCGAAATCGACGGAAG*GCTTCTACACCGACTCTGAAGAC
SK-Sep-119	Antisense	*GGTGAACAGCTCCTCGCCCTTGCTCACCAT*GTTGAAGGTAATGAGGTGGTAAAG
SK-Sep-120	Antisense	*CATTTGCGGCTGTCTCGAAATCGACGGAAG*ATGACAGCTAAACCTTGGGACAG
SK-Sep-121	Antisense	*GGTGAACAGCTCCTCGCCCTTGCTCACCAT*GCTGGGCGTGTGTTGATGGAC
SK-Sep-126	Antisense	*CATTTGCGGCTGTCTCGAAATCGACGGAAG*GTCACCAAGTATGCCGATGACAAC
SK-Sep-127	Antisense	*GGTGAACAGCTCCTCGCCCTTGCTCACCAT*GTCGGGCTTGTGACAGCAGGTG
SK-Sep-136	Sense	CCCAACTGATATTGAAGGAGCATT
SK-Sep-137	Antisense	CCCGATCTAGTAACATAGATGACA
SK-Sep-217	Sense	*TGGCAGGATATATTGTGGTGTAAACAAATT*GCAGTCGACGCCAGATGATGG
SK-Sep-218	Antisense	*CCAAAAAATGCTCCTTCAATATCAGTTGGG*GGCGATGGTGGTATGCGGATG
SK-Sep-221	Sense	ATGCGTGAAGTCATCTCCTTGAAC
SK-Sep-222	Antisense	*TAAACGCTCTTTTCTCTTAGGTTTACCCGC*TGGGACGCTCGATGCCAAGGTT
SK-Sep-225	Sense	*GCGCGGTGTCATCTATGTTACTAGATCGGG*GGGAGAGCTTGCCCGCTGGC
SK-Sep-226	Antisense	*CGTACCGTTCAAGGAGATGACTTCACGCAT*GACTTTGTTTGATCTCGAAGCTGA

a *Italics* indicate part of the primer that is complementary with another DNA fragment, to be ligated by homologous recombination in *S. cerevisiae*.

**Table 2:** Overview of the conditional *Z. tritici* promoters presented in this study.

	Expression under ON conditions^a^	Leakiness under OFF conditions^b^	Induced by	Repressed by
P*nar1*	2	4	Nitrate	Ammonium
P*laraB*	1	3	Arabinose	Glucose
P*ex1A*	2	5	Xylose	Maltodextrin
P*gal7*	3	2	Galactose	Glucose
P*icl1*	5	1	Sodium acetate	Glucose

a 5 = very strong, 4 = strong, 3 = medium, 2 = weak, 1 = very weak.

b 5 = very tight, 4 = low expression, 3 = considerable expression, 2 = strong background expression, 1 = very strong background expression.
